# A point mutation in the zinc-finger transcription factor *CqLOL1* controls the green flesh color in chieh-qua (*Benincasa hispida* Cogn. var. *Chieh-qua* How)

**DOI:** 10.3389/fpls.2024.1388115

**Published:** 2024-10-21

**Authors:** Jiazhu Peng, Yin Gao, Yanchun Qiao, Guoping Wang

**Affiliations:** ^1^ Vegetable Research Science Department, Guangzhou Academy of Agricultural and Rural Sciences, Guangzhou, China; ^2^ Vegetable Research Institute, Guangzhou Academy of Agricultural Sciences, Guangzhou, China; ^3^ College of Horticulture, South China Agricultural University, Guangzhou, China

**Keywords:** chieh-qua, flesh color, chlorophyll content, fine-mapping, zinc-finger transcription factor LOL1

## Abstract

**Introduction:**

Flesh color is an essential trait in chieh-qua (*Benincasa hispida* Cogn. var. *Chieh-qua* How); however, the inheritance and molecular basis of green flesh trait remain unclear.

**Methods:**

In the present study, two F_2_ populations, derived from 1742 (white flesh) × FJ3211 (green flesh) and J16 (white flesh) × FJ5 (green flesh), were used to identify the green flesh (*Cqgf*) locus.

**Results:**

Genetic analysis revealed that the presence of green flesh was a quantitative trait that closely followed a normal distribution. Combining the results from QTL mapping and BSA-seq analysis, the *Cqgf* locus was preliminarily determined to be located on chromosome 05 and was narrowed down to a 2.55-Mb interval by linkage analysis. A large J16 × FJ5 F_2_ population comprising 3,180 individuals was subsequently used to screen the recombinants, and the *Cqgf* locus was fine-mapped to a region of 329.70 kb that harbors six genes. One of the candidate genes, *Bch05G003700*, the zinc-finger transcription factor LOL1 (lsd one like 1 protein; *CqLOL1*), was the strongest candidate gene for the *Cqgf* locus according to sequence variation and expression analysis. Additionally, a point mutation (A > C) in *CqLOL1* resulted in the substitution of threonine (T) with proline (P) in the amino acid sequence, showing a complete relationship linked with flesh color in a panel of 45 germplasms.

**Discussion:**

The study suggests that *CqLOL1* promotes the accumulation of chlorophyll content in chieh-qua and lead to green flesh. Our findings establish a theoretical and technical foundation for breeding different flesh color lines and elucidating the underlying mechanisms of flesh color in chieh-qua.

## Introduction

The color of fruit flesh is a crucial characteristic that holds significant commercial value. It is primarily determined by the content and composition of lycopene, anthocyanin, carotenoids, and chlorophyll, making it a key trait for breeding purposes (Blas et al., 2010; Wang et al., 2019; Zhao et al., 2022). Chlorophyll, composed of chlorophyll a and b, contributes predominantly to green pigmentation and plays a critical role in plant development. It serves as the site of photosynthesis and facilitates the production of other important components such as hormones, amino acids, and fatty acids. Cucurbit crops exhibit a diverse range of flesh colors, including red, yellow, orange, white, and green.

In watermelon (*Citrullus lanatus*), the red flesh is composed mainly of lycopene and a small amount of β-carotene (Kang et al., 2010), orange flesh is largely pigmented by β-carotene (Branham et al., 2017), and the yellow and canary yellow flesh, respectively, results from small amounts of violaxanthin and neochrome (Bang et al., 2010). The genetic basis of flesh color in watermelon is complex, and several loci/genes are known to affect the flesh color of watermelon. The *C* and *I* loci govern canary yellow and red flesh in watermelon (Poole, 1944). In addition, canary yellow (*C*) is dominant over red flesh (*c*) but can be suppressed by homozygous recessive *i* loci which can lead to red flesh regardless of which *C* allele is present (Henderson et al., 1998). The *Cyf* locus for canary yellow flesh color was determined to be a 79.62-kb region on chromosome 6 harboring 10 predicted genes, and *Cla97C06G122050* and *Cla97C06G122120* (pentatricopeptide repeat protein) were predicted to be important candidate genes (Liu et al., 2023). Compared with canary yellow flesh, white flesh is controlled by a recessive locus, and this locus (*Clwf2*) was determined to be located within a 132.3-kb region on chromosome 6 which included 13 candidate genes (Yi et al., 2023). A locus associated with pale yellow and white flesh color was mapped on chromosome 6 and further narrowed into a 66.8-kb region containing nine candidate genes, of which *Cla007528*, which has a non-synonymous mutation, was suggested to be the strongest candidate gene (Wang et al., 2021). A major locus responsible for β-carotene accumulation was determined on chromosome 1 through linkage analysis (Branham et al., 2017). Red flesh color is recessive to pale yellow color, and the locus was narrowed to a 24-kb region on chromosome 4 harboring two genes, of which the lycopene β-cyclase gene (*LCYB*) was considered the key gene (Wang et al., 2019). Furthermore, the downregulation of *LCYB* leads to a change in flesh color from pale yellow to red, while the overexpression of *LCYB* in red flesh lines leads to a change to orange (Zhang et al., 2020). Genome-wide association studies have also shown that the mutation of the *LCYB* gene from phenylalanine to valine leads to increased lycopene accumulation, and other candidate gene *ClTST2* on chromosome 2 likely promotes carotenoid accumulation through *ClPHT4* (Guo et al., 2019). In addition, the expression of *ClPHT4* was necessary for carotenoid lycopene accumulation (Zhang et al., 2016), and *ClPSY1* was positively responsible for lycopene accumulation (Guo et al., 2019). The scarlet red flesh (*Y^scr^*) was dominant over coral red flesh in watermelon and was located on chromosome 6 within a 40-kb interval which included five putative genes (Li et al., 2020). In addition, a major QTL (*qfc10.1*) related to pale green flesh color and chlorophyll content was found within a 519-kb region on chromosome 10 harboring 22 annotated genes (Pei et al., 2021).

In melon (*Cucumis melo L.*), due to the accumulation of chlorophyll and carotenoids, the flesh color mainly appears light green, white, or orange, and β-carotene is the predominant pigment (Shahwar et al., 2023). In previous studies, the genetic basis of flesh color variation was partly resolved, and two major genes, green flesh (*gf*) and white flesh (*wf*), were shown to govern the flesh color. *CmOr*, located on chromosome 9 and previously described as the *gf* locus in melon, had two haplotypes (alleles), one of which was associated with orange flesh and the other was associated with white or green flesh (Tzuri et al., 2015). The SNP within *CmOr* was also confirmed by complete segregation with orange flesh (Gur et al., 2017). The *wf* locus was likely to be *CmPPR1* (*MELO3C003069*), which was located on chromosome 8. This gene encodes pentatricopeptide protein and participates in RNA processing in the plastid where carotenoids and chlorophyll accumulate (Galpaz et al., 2018). To decipher the *wf* locus, a 96-kb-overlap interval containing 11 protein coding genes was determined by QTL and GWAS, which excluded the *CmPPR1* gene, but *MELO3C003097* was confirmed to be a strong candidate gene (Zhao et al., 2019). In addition, *CmAPRR2* was identified as a causal gene for pigment content in the rind and flesh of mature melon fruits (Oren et al., 2019).

Most of the cucumber flesh is white, while some materials have orange or green flesh because of the accumulation of β-carotene and chlorophyll. Compared with that in white flesh, the β-carotene content in the mesocarp was controlled by two recessive genes (Cuevas et al., 2010), and the β-carotene content in the endocarp was controlled by a recessive gene (Bo et al., 2012). The locus associated with orange flesh in the endocarp (*CsOr*) was subsequently identified on chromosome 6, which included two carotenoid-related genes, of which *Csa6G452720*, homolog of *CmOr*, was considered the causal gene of orange flesh (Kishor et al., 2021). Green flesh (*Csgf*) seems to be incompletely recessive to white flesh, and *Csgf* was controlled by two QTLs, *qgf5.1* and *qgf3.1* (Bo et al., 2019). The yellow flesh (*yf*) locus was fine-mapped into a 150-kb region on chromosome 7 harboring 21 candidate genes (Lu et al., 2015).

Chieh-qua (*Benincasa hispida* Cogn. var. *Chieh-qua* How), one of the most important cucurbit vegetables, is classified as a variety of wax gourd (*Benincasa hispida* (Thunb.) Cogn.) and is extensively cultivated in southern China and various Southeast Asian countries. Unlike the mature stage consumption of wax gourd, chieh-qua fruits can be harvested at both immature and mature stages, depending on market demands, and mature fruits have the advantage of long-term storage. In contrast to those of other cucurbits, the flesh color of chieh-qua can be categorized into two primary types: green and white. However, the genetic basis underlying flesh color in chieh-qua has not been determined. Since the completion of wax gourd genome sequencing (Luo et al., 2023; Xie et al., 2019), a scientific foundation has been established to unravel the molecular regulatory mechanisms governing traits, including flesh color.

This study aimed to elucidate the regulatory gene responsible for the green flesh trait in chieh-qua. To achieve this, two F2 populations with different genetic backgrounds were utilized for the genetic analysis of green flesh. QTL mapping, BSA-seq, and fine-mapping were performed to identify the *Csgf* locus. By combining expression and sequence variation analysis, we identified an important candidate gene *Bch05G003700*, namely *CqLOL1*, which belongs to the zinc-finger transcription factor LOL1 family. Our findings not only facilitate marker-assisted selection but also enhance our understanding of the molecular mechanisms underlying green flesh development.

## Materials and methods

### Plant materials and phenotypic evaluation

To generate segregating populations for analysis, two white flesh lines, 1742 and J16, were crossed with two green flesh lines, FJ3211 and FJ5. The resulting populations included 120 individuals in the 1742 × FJ3211 F_2_ population and 363 individuals in the J16 × FJ5 F_2_ population. Two distinct sets of materials were utilized to investigate the inheritance patterns of green flesh. Specifically, the 1742 × FJ3211 F2 population was employed for the construction of genetic maps and for conducting QTL mapping, while the J16 × FJ5 F2 population was utilized for BSA-seq, linkage mapping, and precise mapping of the candidate genes associated with green flesh. The flesh of J16 and FJ5 were sampled at 5, 10, 15, 20, 25, and 30 days after pollination (DAP), and the flesh color was significantly different at all stages (**Supplementary Figure S1**). To conduct shading experiments, light-impermeable bags were employed to completely cover the female flower of FJ5, including the ovary, after pollination. Additionally, the flesh sampled at 10 DAP under shading conditions were collected to analyze the expression of candidate genes.

In addition, 45 extreme chieh-qua materials were used to clone and compare the causal gene controlling the flesh color and used for marker-assisted selection tests. All the materials were provided by Guangzhou Academy of Agricultural Sciences and cultivated at the Nansha Experimental Base of the Guangzhou Academy of Agricultural Sciences (Guangzhou, China, 23.4 N, 113.4 E). The phenotype of flesh was evaluated by two indices at 10 DAP, flesh color grouping (FCG), and flesh chlorophyll content (FCC). FCG was categorized into four types by visual inspection, and FCC was extracted with 95% alcohol and calculated by using a previous method (Bo et al., 2019).

### QTL mapping analysis

The genotypes of F_2_ individuals derived from 1742 × FJ3211 were obtained through SLAF-seq (Sun et al., 2013), and a high-density genetic map (unpublished) was constructed by using a method developed previously (Li et al., 2008). The map was divided into 12 linkage groups containing 5,354 markers and spanned a total of 1,997.32 cM, with an average distance of 0.37 cM between adjacent markers. QTL mapping was conducted using Highmap software via the composite interval mapping (CIM) method, and the maximum limit of detection (LOD) threshold was determined via permutations (1,000 times) (Liu et al., 2014).

### BSA-seq analysis

The green flesh (GF) and white flesh (WF) DNA pools were constructed by mixing equal amounts of DNA samples from 30 green and 30 white flesh F_2_ individuals derived from J16 × FJ5, respectively. The two extreme bulks were subjected to whole-genome resequencing on the Illumina HiSeq platform of Biomarker Technologies (www.biomarker.com.cn). The resequencing data were filtered and aligned to the reference genome of wax gourd (GX-19; unpublished) using BWA software with default parameters. Then, the variations including InDels and SNPs were detected by using GATK software, and high-quality InDels and SNPs with GQ>50 in the two bulks were subjected to InDel and SNP index analysis. Then, the ΔInDel-index and ΔSNP-index were obtained by using the InDel and SNP index algorithms, respectively, for the two bulks by sliding window analysis with a 200-kb window size and a step length of 100 kb, and the fitted values with 99% confidence interval were used to identify the candidate region responsible for the green flesh. After overlapping the regions identified by two algorithms, we obtained the final candidate interval governing green flesh from BSA-seq.

### Genotyping and linkage mapping analysis

The resequencing data of materials J16 and FJ5 were also aligned to the reference genome of wax gourd, and eight polymorphic InDel markers, Fc_1–Fc_8, within the candidate region were developed to genotype their 363 F2 individuals using Primer 3plus (https://primer3.ut.ee/). A molecular linkage map was constructed using IciMapping software (Meng et al., 2015). Polymerase chain reaction (PCR) was conducted in a 10-µL reaction mixture containing 1 µL of the DNA template (50–100 ng µL^−1^), 1 µL of forward primer (10 µmol L^−1^) and reverse primer (10 µmol L^−1^), 5 µL of 2× GoTaq Green Master Mix, and 2 µL of ddH_2_O. The PCR procedure was as follows: 95°C for 3 min; 34 cycles of 95°C for 30 s, 55°C for 30 s, and 72°C for 30 s; and 72°C for 5 min. The PCR products were subjected to 6% polyacrylamide gel eletrophoresis (PAGE). To narrow the region of *Cqgf*, a large J16 × FJ5 F_2_ population consisting of 3,810 individuals was genotyped by two flanking markers, Fc1 and Fc5, for screening of homozygous recombinants. Furthermore, five InDel markers, Fc_9–Fc_13, within the region were developed to genotype the recombinants. All InDel markers are listed in **Supplementary Table S2**. Then, we constructed genotype-based haplotypes for the recombinants and inferred the most likely region of *Cqgf*.

### Sequence variation analysis and dCAPS marker development

Total RNA was extracted from the flesh using the Eastep Super Total RNA Extraction Kit (Promega, Shanghai, China), and cDNA was obtained by reverse transcription using a cDNA synthesis kit (Promega, Shanghai, China). Candidate genes responsible for the green flesh were cloned and subjected to Sanger sequencing by Beijing Tsingke Biotech Co., Ltd. Sequence alignment was performed using DNAMAN software.

### Expression analysis

The expression of candidate genes was assessed in different tissues including root, stem, leaf, and flesh at six different stages (5 to 30 DAP) and the 10 DAP flesh under shading using quantitative real-time polymerase chain reaction (qRT-PCR). The expression levels of three biological and technical repeats for each sample were calculated with the ΔΔCt method (Livak and Schmittgen, 2001). The primer pairs are shown in **Supplementary Table S4**.

### Construction of the phylogenetic tree

The homologous genes of LOL1 in different crops were searched from NCBI database to obtain the corresponding amino acid sequences. Then, all of the amino acid sequences were aligned by using MUSCLE, and a neighbor-joining phylogenetic tree was constructed using MEGA11.0 with 1,000 bootstrap replications. Finally, the phylogenetic tree and predicted motif were integrated and drawn with TBtools (Chen et al., 2023).

## Results

### Phenotypic analysis of green flesh

**Figure 1:**
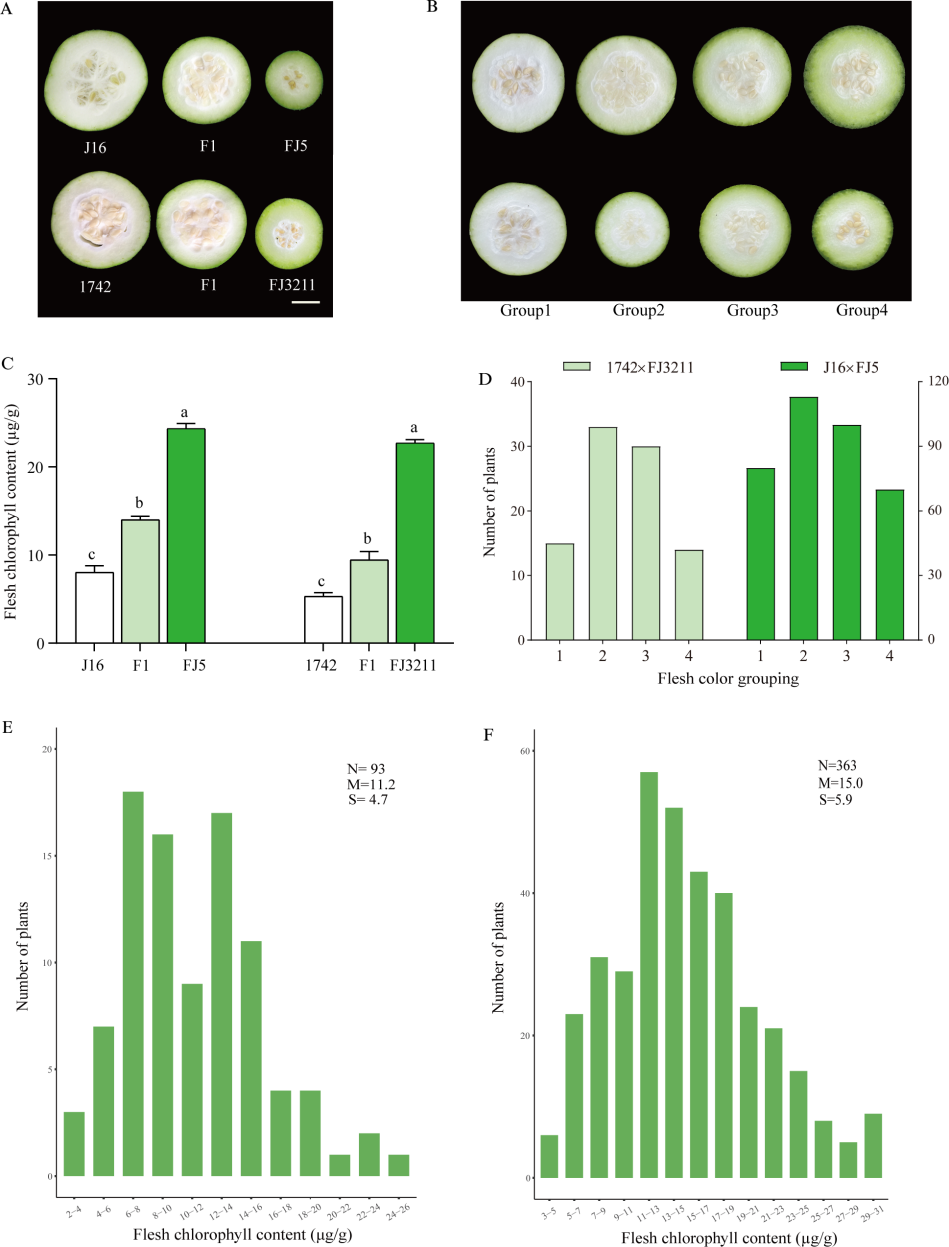
Flesh color and chlorophyll content of the four parents, their F_1_ and F_2_ generations. **(A)** Flesh color performances of four parents and their F_1_, scale bar = 3 cm. **(B)** Flesh color of the four groups in F_2_ population. **(C)** Flesh chlorophyll content of four parents and their F_1_. The chlorophyll contents are the mean ± SD (*n* = 3). **(D)** Flesh color grouping distributions of two F_2_ populations. **(E)** Flesh chlorophyll content distribution of the F_2_ population derived from 1742 × FJ3211. **(F)** Flesh chlorophyll content distribution of the F_2_ population derived from J16 × FJ5. a,b,c represent significant difference at the 0.05 level; N, the number of investigated plants; M, mean; S, standard deviation.

To identify the inheritance pattern of flesh color, two white flesh (1742 and J16) and two green flesh (FJ3211 and FJ5) chieh-qua inbreds were used to construct two segregating populations, 1742 × FJ3211 and J16 × FJ5 F_2_ population, and the flesh of parents and their F1 are shown in **Figure 1A**. The phenotypes of FCG were categorized based on visual observation (**Figure 1B**), and FCC were collected from two populations, including two panels of parents, their F_1_, and 93 and 363 individuals from 1742 × FJ3211 and J16 × FJ5 population, respectively. Significant differences in FCC were observed among the female parent, male parent, and F_1_ progeny. Notably, two male parents exhibited the highest FCC values, and their F_1_ progeny displayed a biased preference toward white flesh (**Figure 1C**). The frequency distributions of FCG and FCC from both crosses were continuous, suggesting the quantitative nature of flesh color (**Figures 1D–F**).

### QTL mapping analysis

**Figure 2:**
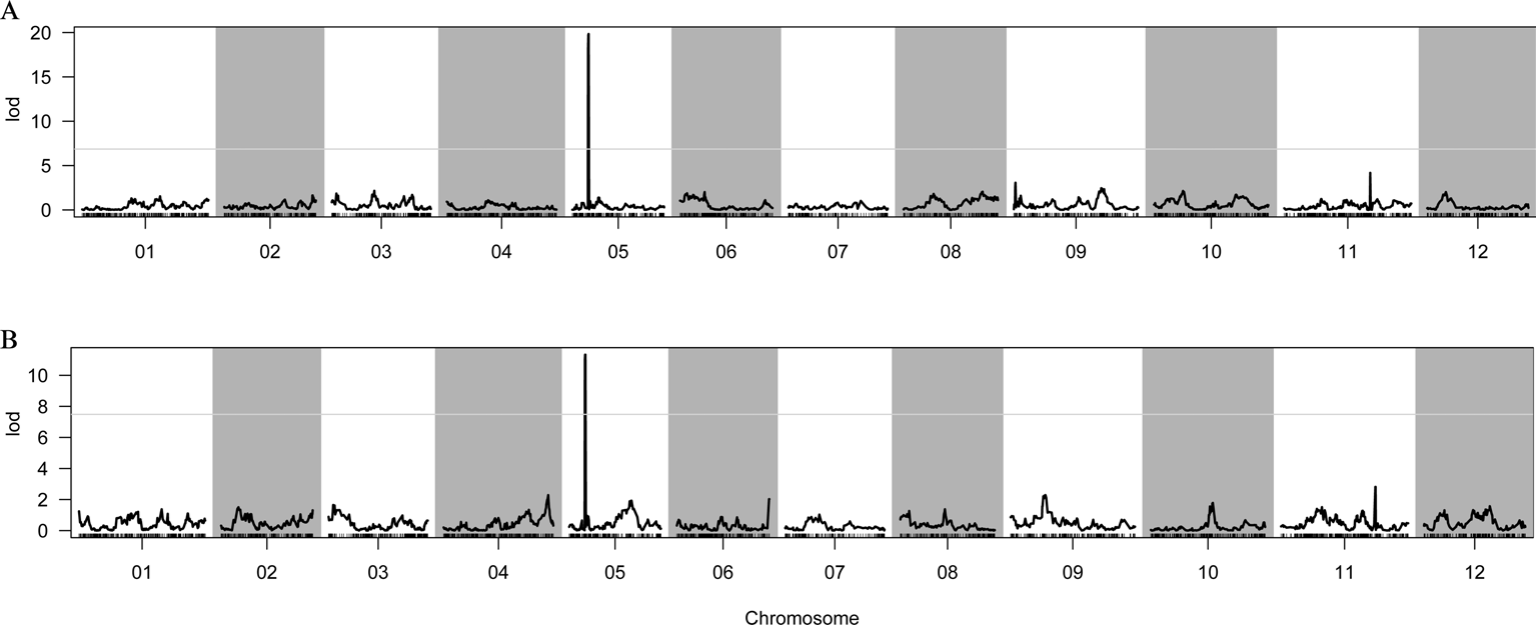
Genetic mapping of FCG and FCC by the 1742 × FJ3211 F_2_ population. **(A)** Distribution of LODs of FCG. **(B)** Distribution of LOD values of FCC. 01–12 represent the linkage groups corresponding to the chromosomes of the reference genome.

A high-density genetic map was generated using sequencing data obtained from the F_2_ population of 1742 × FJ3211. Subsequently, a QTL analysis was conducted using the composite interval mapping (CIM) method, employing FCG and FCC indexes. The results revealed the presence of a single peak for both FCG and FCC on chromosome 05 (**Figures 2A, B**). Notably, these two QTLs, designated *qfcg5* and *qfcc5*, were consistently located at 24.67–25.51 cM and exhibited LODs of 19.84 and 11.33, respectively (**Table 1**). These QTLs accounted for 48.62% and 38.75% of the phenotypic variation, respectively, suggesting that a major locus governs the green flesh trait.

**Table 1:** Quantitative trait loci (QTLs) for flesh color in the F_2_ population.

Trait	QTL	Chromosome	Position interval (cM)	LOD	PVE(%)	ADD	Physical interval
FCG	*qfcg5*	5	24.67–25.51	19.84	48.62	-0.96	11548007–15769121
FCC	*qfcc5*	5	24.67–25.51	11.33	38.75	-4.07	11548007–15769121

### BSA-seq identified the *Cqgf* locus

**Figure 3:**
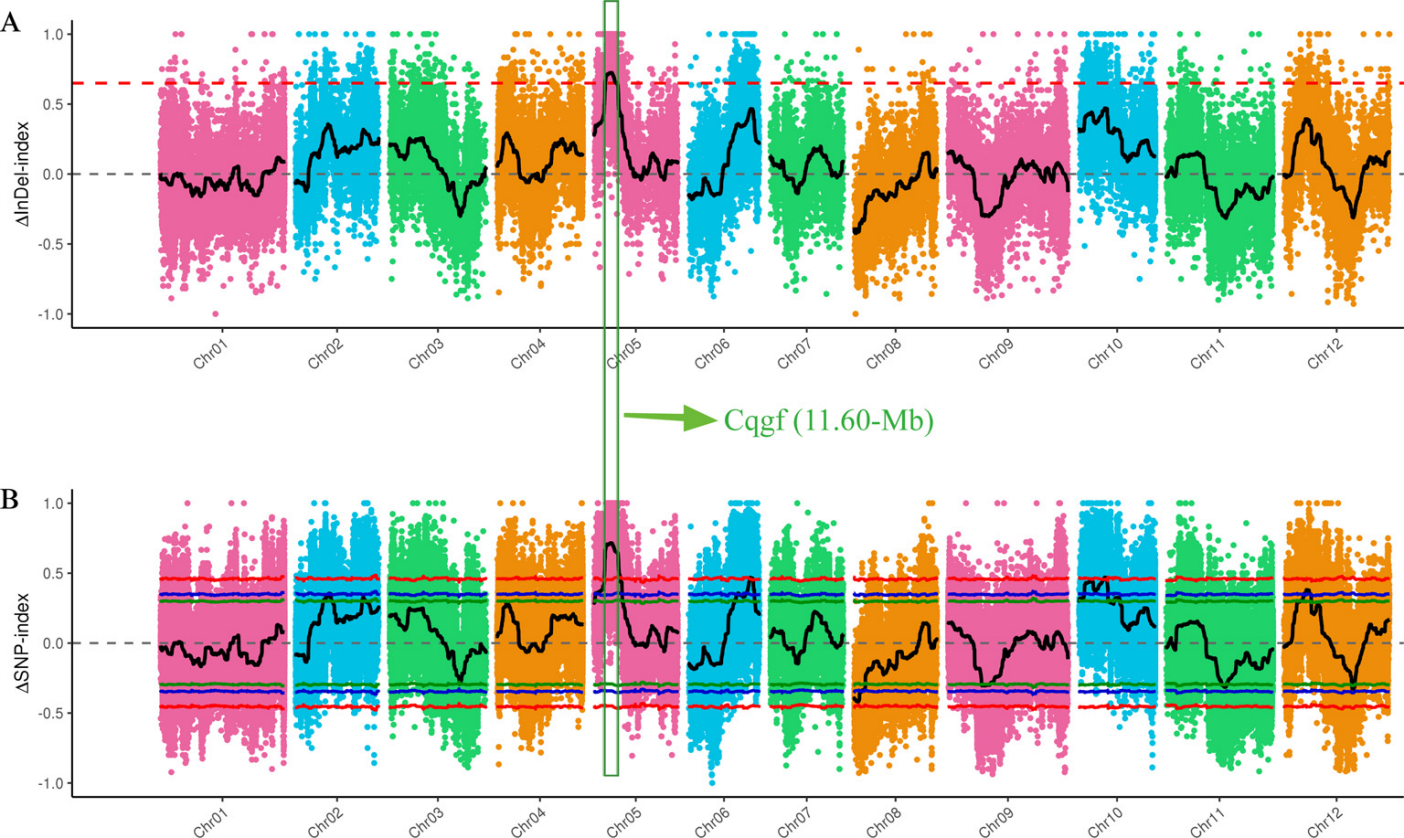
BSA-seq mapping of the *Cqgf* locus. **(A)** InDel index algorithm for two bulks. **(B)** SNP index algorithm for two bulks. The overlapping region is surrounded by the green box.

After filtering the raw reads from the whole-genome resequencing of the two extreme bulks (GF and WF), 2.03 billion (~30.32 Gb) and 2.12 billion (~31.67 Gb) clean reads were generated, with Q20 values of 98.95% and 98.86%, respectively (**Supplementary Table S1**). Then, a large number of high-quality InDels and SNPs were identified through variant calling and used to identify the *Cqgf* locus. The application of the InDel index algorithm to two bulks identified a prominent locus on Chr05 spanning from 8.56 to 20.06 Mb (**Figure 3A**). Furthermore, the employment of the SNP index algorithm with two bulks revealed three distinct loci. One of these regions was situated on Chr05, covering the genomic interval from 8.32 to 21.40 Mb (**Figure 3B**). The remaining two regions were located on chr06 (50.30 to 52.76 Mb) and chr10 (20.68 to 22.94 Mb). Overlapping the regions identified by two algorithms, *Cqfc* locus was preliminarily located on Chr05 from 8.56 to 20.06 Mb, with a physical distance of 11.60 Mb.

### Fine-mapping of the *Cqgf* locus

**Figure 4:**
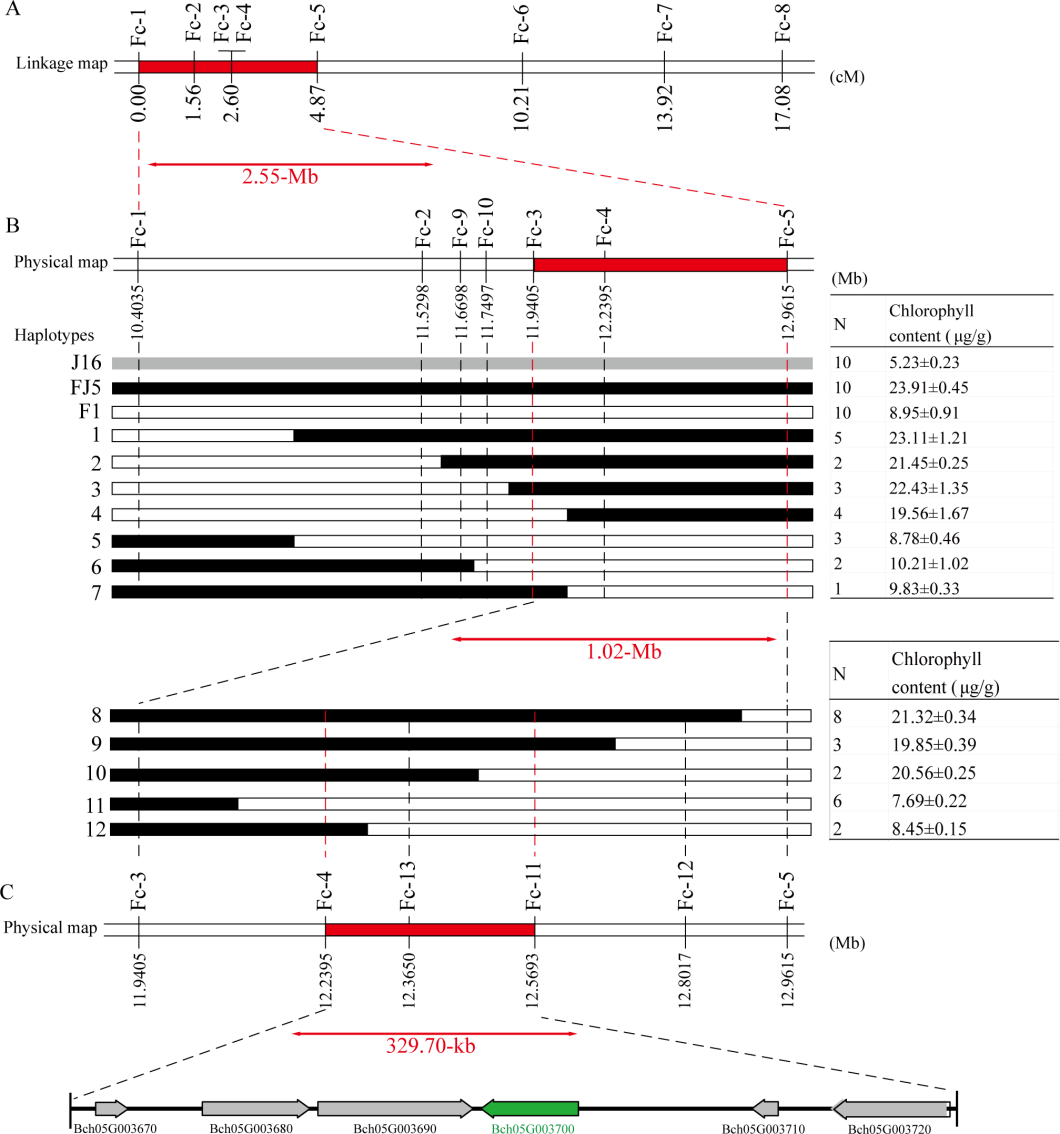
Fine mapping of the *Cqgf* locus. **(A)** Linkage mapping of the *Cqgf* locus. The number below the bars represents the genetic distance. The *Cqgf* locus is located in the region highlighted in red color and flanked by markers Fc_1 and Fc_5. **(B)** Physical map of the markers used to genotype the 20 recombinant plants. The number below the bar corresponds to the physical position of the reference genome. Seven haplotypes (1–7) representing the 20 recombinant plants screened (with markers Fc_1 and Fc_5) from 781 F_2_ individuals and five haplotypes (8–12) representing the 21 recombinant plants screened (with markers Fc_3 and Fc_5) from 2,399 F_2_ individuals. The red bar represents the *Cqgf* locus. **(C)** Physical map of the markers used to genotype the 21 recombinant plants. The arrows represent the predicted genes.

After integrating the findings from genetic mapping and BSA-seq, the *Cqgf* locus was determined to be located on Chr05. Given that the *Cqgf* locus was previously identified through analysis of diverse segregating populations, the 8.58–20.18-Mb region, which encompasses the region identified in the genetic mapping analysis, was selected as the locus for subsequent investigations. To further verify the *Cqgf* locus, eight polymorphic InDel markers, Fc_1–Fc_8, distributed within the candidate region were developed and used to genotype 363 individuals from the J16 × FJ5 F_2_ population. Based on the genotypes and phenotypes, a molecular linkage map with a genetic distance ranging from 0 to 17.08 cM was constructed, and *Cqgf* was narrowed to a 2.55- Mb region from 10.40 to 12.96 Mb flanked by the InDel markers Fc_1 and Fc_5 (**Figure 4A**).

Based on genotyping with the two flanking markers (Fc_1 and Fc_5), 20 recombinant plants were identified from the 781 F_2_ individuals. Then, those recombinants were divided into seven haplotypes using the markers Fc_2–Fc_4 and two newly developed markers (Fc_9 and Fc_10). By utilizing the chlorophyll content and genotype data of recombinants, the *Cqgf* locus was further mapped into a 1.02-Mb physical interval flanked by the markers Fc_3 and Fc_5 (**Figure 4B**).

To determine a more precise region for the *Cqgf* locus, a large J16 × FJ5 F_2_ population consisting of 2,399 individuals was genotyped by the markers Fc_3 and Fc_5, and a total of 21 homozygous recombinants were obtained. Using the Fc_4 marker and three newly developed markers (Fc_11–Fc_13), those recombinants were divided into five haplotypes. Notably, the marker Fc_13 showed a complete linkage relationship with the chlorophyll content of 21 recombinants. Thus, the *Cqgf* locus was finally fine-mapped to a 329.7-kb region from 12.24 to 12.57 Mb (**Figure 4C**). Based on the genome of wax gourd, six genes were predicted (**Table 2**). The genes *Bch05G003680*, *Bch05G003690*, *Bch05G003700*, and *Bch05G003720* were found to encode the phospholipid hydroperoxide glutathione peroxidase, 2Fe-2S ferredoxin-like protein, lsd one like 1 protein, and plant intracellular ras-group-related LRR protein 4, respectively. The remaining two genes were found to encode uncharacterized proteins.

**Table 2:** List of six annotated genes for the *Cqgf* locus.

GeneID	Start	End	Ortholog in *Arabidopsis thaliana*	Gene symbol	Anotation description	Score (bits)	E-value
Bch05G003670	12289386	12290252	–	–	Uncharacterized protein	–	–
Bch05G003680	12358903	12361768	AT4G17960	AT4G17960	Phospholipid Hydroperoxide glutathione peroxidase	164	1.00E-52
Bch05G003690	12364349	12368482	AT1G32550	FdC2	2Fe-2S ferredoxin-like superfamily protein	267	2.00E-92
Bch05G003700	12375449	12378043	AT1G32540	LOL1	lsd one like 1 protein	219	2.00E-74
Bch05G003710	12519688	12520389	–	–	Reverse transcriptase	–	–
Bch05G003720	12565673	12568763	AT4G35470	PIRL4	Plant intracellular ras-group-related LRR protein 4	454	4.00E-155

### Expression analysis of candidate genes by qRT-PCR

**Figure 5:**
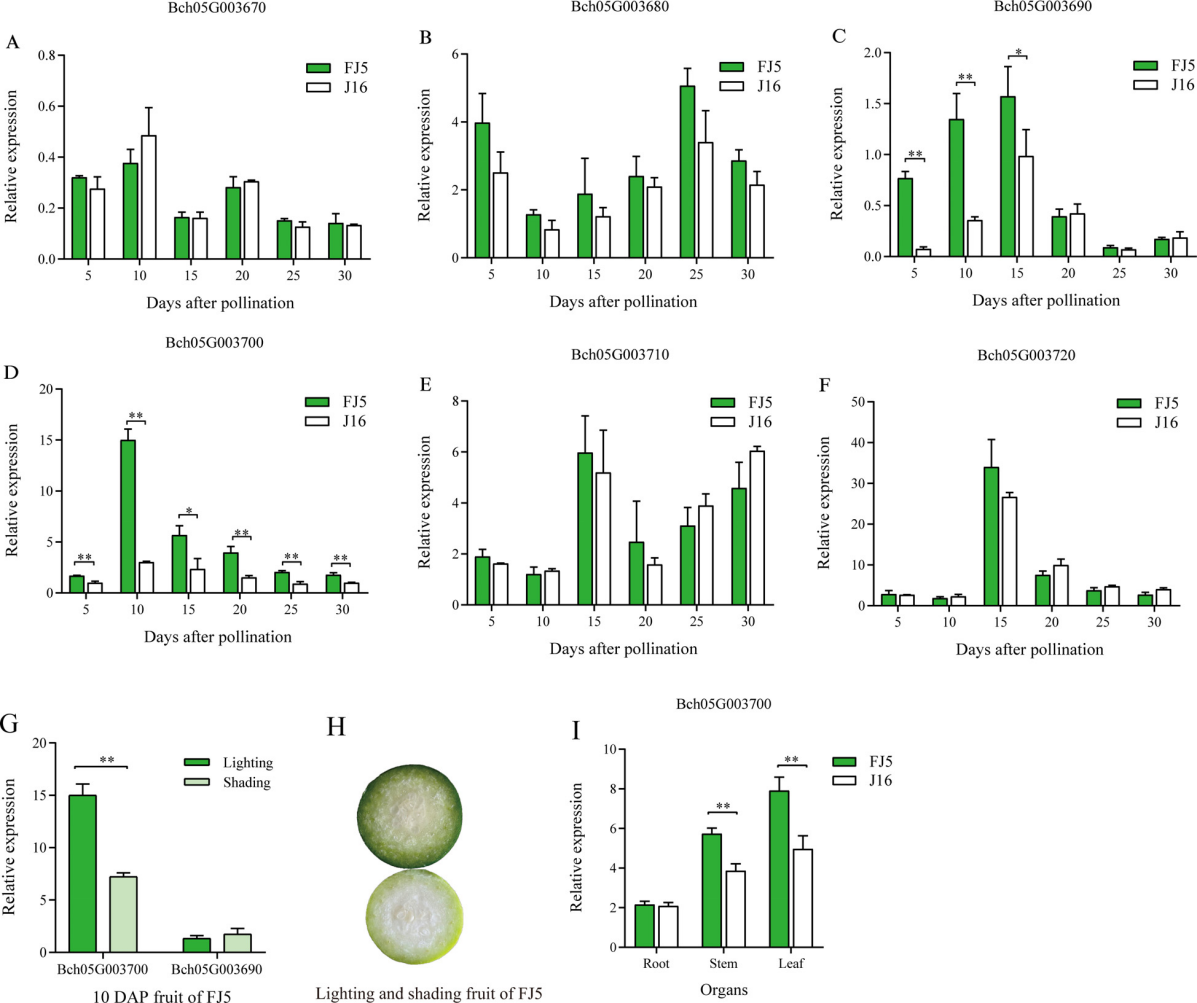
Expression analysis of the candidate genes between FJ5 and J16. **(A)** The relative expression levels of *Bch05G003670* from 5 to 30 DAP. **(B)** Relative expression levels of *Bch05G003680* from 5 to 30 DAP. **(C)** Relative expression levels of *Bch05G003690* from 5 to 30 DAP. **(D)** Relative expression levels of *Bch05G003700* from 5 to 30 DAP. **(E)** Relative expression levels of *Bch05G003710* from 5 to 30 DAP. **(F)** Relative expression levels of *Bch05G003720* from 5 to 30 DAP. **(G)** Expression levels of *Bch05G003690* and *Bch05G003700* in 10 DAP flesh of FJ5 under lighting and shading condition. **(H)** Phenotypes of the 10 DAP flesh in FJ5 under lighting and shading condition. The upper part of fruit was under normal lighting and the below part was under shading. **(I)** Expression levels of *Bch05G003700* in root, stem, and leaf. The relative expression levels are mean ± SD (*n* = 3). * represents significance at the 0.05 level (Student’s *t*-test), ** represents significance at the 0.01 level (Student’s *t*-test).

To further determine the causal gene of flesh color, we compared the relative expression levels of the six candidate genes from 5 to 30 DAP. The results demonstrated that the expression levels of the four genes, *Bch05G003670*, *Bch05G003680*, *Bch05G003710*, and *Bch05G003720*, did not exhibit significant differences across all developmental stages (**Figures 5A, B, E, F**). Remarkably, the expression levels of *Bch05G003700* were significantly higher than that in J16 at six stages, which corresponded to the observed difference in flesh color of the parents (**Supplementary Figure S1**), reaching its peak at 10 DAP and subsequently decreasing (**Figure 5C**). Similarly, the expression level of *Bch05G003690* in FJ5 was significantly higher than that in J16 at 5, 10, and 15 DAP, whereas no significant difference was observed from 20 to 30 DAP (**Figure 5D**).

The homologs of these two genes were associated with chlorophyll synthesis in other species. Specifically, *Bch05G003690*, classified as a ferredoxin-like protein, played a crucial role in electron transfer during chlorophyll biosynthesis, and *Bch05G003700*, a zinc-finger transcription factor LOL1, was identified as a regulator of programmed cell death in *Arabidopsis* and rice (Epple et al., 2003; Wang et al., 2005) and as a regulator of controlled chloroplast development in tomato and pepper (Borovsky et al., 2019). Thus, these two genes were most likely causal genes associated with green flesh in chieh-qua. Furthermore, as the green flesh color was lighter under shading conditions (**Figure 5H**), the 10 DAP flesh of FJ5 under shading was used to evaluate the impact of shading on the expression levels of *Bch05G003690* and *Bch05G003700*. The results showed that there was no significant alteration in the expression level of *Bch05G003690* between lighting and shading conditions (**Figure 5G**). In contrast, the expression level of *Bch05G003700* significantly decreased under shading conditions and reached approximately half of the expression level observed under lighting conditions (**Figure 5G**). The reduced expression level of *Bch05G003700* corresponded to the observed phenotypic difference. In addition, *Bch05G003700* gene was also highly expressed in leaf and stem, indicating that *Bch05G003700* was highly expressed in green tissue, and there was a significant difference in the expression of stem and leaf between two parents (**Figure 5I**).

### Sequence characterization of the candidate genes

**Figure 6:**
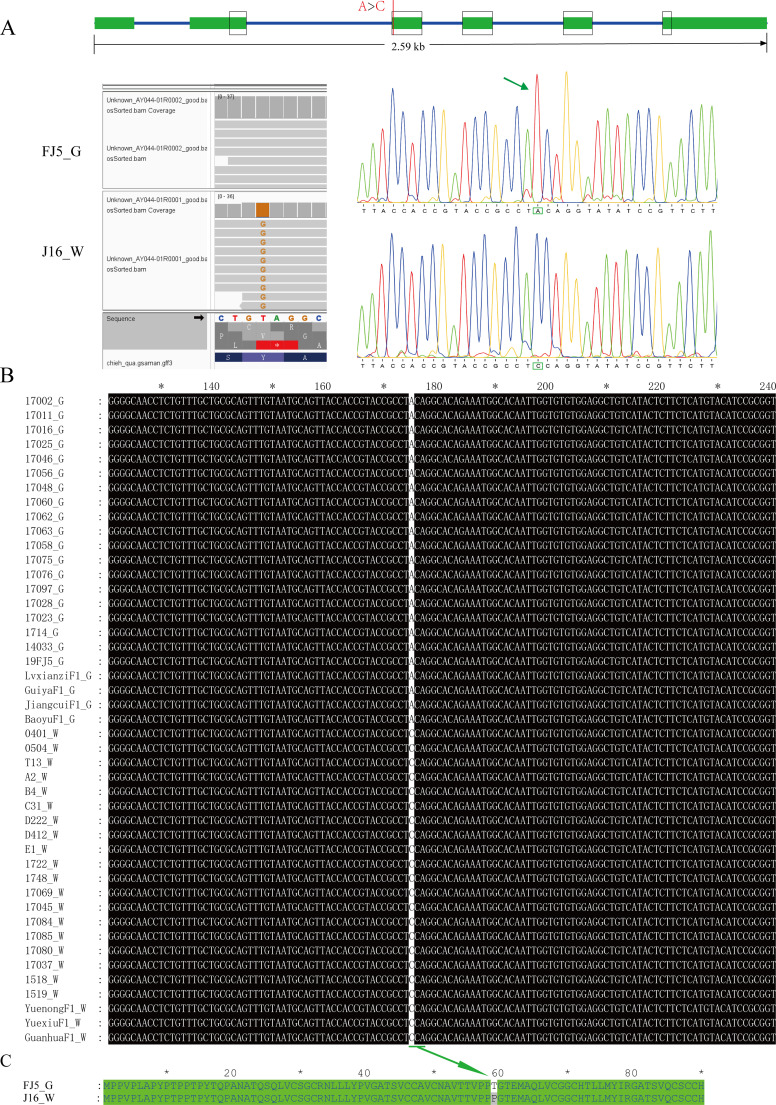
Variation of the nucleotide and amino acid sequences of *Bch05G003700*. **(A)** Gene structure and variation position between J16 and FJ5. **(B)** Nucleotide variation in *Bch05G003700* in 45 chieh-qua germplasms. **(C)** Corresponding amino mutational loci. “G” and “W” represent green and white flesh color, respectively.

We then focused mainly on the variation in the coding sequences of *Bch05G003690* and *Bch05G003700* based on the resequencing data of J16 and FJ5. As a result, no variation was detected in the coding sequence of *Bch05G003690* (**Supplementary Figure S2**), while *Bch05G003700* had a point mutation (A>C) in the coding sequence, resulting in the conversion of the amino acid T to P (**Figures 6A, C**). The mutation in *Bch05G003700* was also confirmed through cDNA cloning and Sanger sequencing (**Figure 6A**). Moreover, a perfect correlation between the mutation and flesh color was observed among 45 chieh-qua materials, which had 23 green flesh samples and 22 white flesh samples, including inbred lines and commercial F1 varieties (**Figure 6B**; **Supplementary Table S5**). Taken together, we speculate that *Bch05G003700*, *CqLOL1*, was the strongest candidate gene for the *Cqgf* locus, and the mutation might be responsible for the accumulation of chlorophyll content.

Furthermore, this mutation was developed into a dCAPS marker, named GF-dCAPS, by introducing a mismatch base (G) at the end of the forward primer to create a AgeI restrictive enzyme recognition site. The 45 chieh-qua materials were genotyped with the marker, and the result showed a complete correlation with flesh color (**Supplementary Figure S3**). In addition, the InDel marker Fc_13 was cosegregated with *Cqgf* in all recombinants, so Fc_13 was used to genotype the 45 chieh-qua materials, which also showed a complete accuracy (**Supplementary Figure S4**).

### Phylogenetic analysis

**Figure 7:**
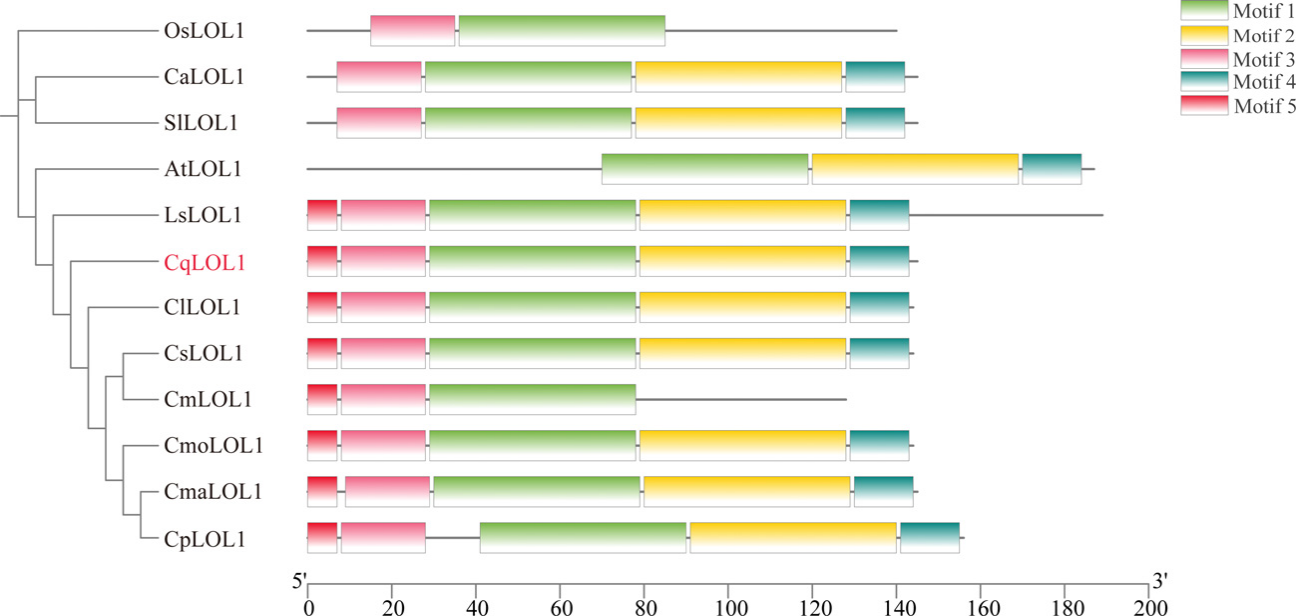
Phylogenetic analysis and conceptual motif information for LOL1 in chieh-qua and other species. The phylogenetic tree and motif distribution of LOL1 from *Oryza sativa* (Os), *Arabidopsis* (At), *Capsicum annuum* (Ca), *Solanum lycopersicum* (Sl), *Cucumis melon* (Cm), *Cucumis lanatus* (Cl), *Lagenaria siceraria* (Ls), *Cucumis sativus* (Cs), *Cucurbita moschata* (Cmo), *Cucurbita maxima* (Cma), and *Cucurbita pepo* (Cp) are shown. The green, yellow, pink, blue, and red bars represent different predicted motifs.

To analyze the relationship between *CqLOL1* and its homologous protein, we constructed a phylogenetic tree through neighbor-joining. The phylogenetic tree showed that LOL1 was conserved in different plant species, and *CqLOL1* was closely related to Cucurbitaceae (**Figure 7**).

## Discussion

Fruit flesh color, an essential feature for consumer preference, is considered a key trait for breeding. To our knowledge, chieh-qua exhibits white and green flesh, and green flesh is significantly associated with soluble sugar content (unpublished data). Genetic analysis indicated that green flesh was a quantitative trait nearly related to normal distribution. To decipher the molecular mechanism of green flesh formation, only one peak, named *Cqgf*, was determined on chromosome 5 through QTL mapping and BSA-seq and was consistent with the major QTL for wax gourd flesh color (*fc5.1*) (Su et al., 2022). Furthermore, based on the large J16 × FJ5 F_2_ population, *Cqgf* was ultimately fine-mapped into a small region (329.70 kb).

Several genes or loci related to green flesh have been identified in cucumber, watermelon, and melon. The green flesh of immature cucumber resulting from the accumulation of chlorophyll was governed by two loci (*qgf5.1* and *qgf3.1*), of which *qgf5.1* was the major-effect QTL, and the gene *Csa5G021320*, which encodes Sec14 cytosolic factor, was a key candidate gene (Bo et al., 2019). In watermelon, a major-effect QTL (*qfc10.1*) related to pale green flesh and chlorophyll content was found within a 519-kb region harboring 22 genes, and the most important candidate gene, *Cla97C10G185970*, encodes a plastid lipid-associated protein and participates in the photoprotection of photosystem II (Pei et al., 2021). In melon, the *wf* locus controls white and green flesh color, and two closely related genes, *MELO3C003069* and *MELO3C003097*, were both reported to be causal genes. *MELO3C003069* belongs to the pentatricopeptide protein family and participates in the processing of RNA in plastids (Galpaz et al., 2018), while the ortholog of *MELO3C003097* in *Arabidopsis* (*SG1*) has been identified to be essential for chloroplast development and chlorophyll biosynthesis (Zhao et al., 2019). According to our results, the *Cqgf* region contains six protein-coding genes based on the annotation of the reference genome GX-19. Unfortunately, none of these genes were homologous to the regulatory genes related to the green color of cucurbits mentioned above. However, both the homologous genes of *Bch05G003690* and *Bch05G003700* have been shown to be required for chlorophyll biosynthesis in other crops. *Bch05G003690* encodes ferredoxin-like proteins with a C-terminal extension (*FdC2*) that was involved in electron transfer, and the mutation of *FdC2* resulted in decreased chlorophyll content in the leaf of *Arabidopsis* (Tournaire et al., 2023) and rice (Li et al., 2015). The other gene, *Bch05G003700*, encodes a zinc-finger transcription factor LOL1 (lsd one like 1), which is highly conserved in different plant species—for example, *AtLOL1* positively regulated programmed cell death in *Arabidopsis* (Epple et al., 2003), and overexpression of *OsLOL1* in rice increased the content of chlorophyll (Wang et al., 2005). The downregulation of *CcLOL1* with a single C nucleotide insertion caused a decrease in chlorophyll content in pepper fruit, and *CcLOL1* was highly expressed in green tissue (Borovsky et al., 2019). The *SlLOL1* knockout mutant in tomato resulted in light green immature fruits (Borovsky et al., 2019).

We then conducted an expression analysis of six candidate genes within the fine-mapping region, especially *Bch05G003690* and *Bch05G003700*. However, *Bch05G003690* and *Bch05G003700* both exhibited significantly different expression levels between J16 and FJ5. Remarkably, *Bch05G003700* exhibited significance from 5 to 30 DAP, which was accordant with the phenotypic differences at six stages. In addition, the flesh color of chieh-qua is influenced by the strength of light, so we conducted shading experiments, and the green flesh clearly turned to pale green under shading (**Figure 5H**). Compared with that under normal lighting conditions, the expression level of *Bch05G003700* under shading was lower, indicating that *Bch05G003700* was induced by light, while there was no significant change in *Bch05G003690* (**Figure 5G**). We subsequently compared the coding sequence variation of candidate genes, and there is no variation in *Bch05G003690*. However, there is a point mutation (A>C) in the coding sequence of *Bch05G003700*, and the amino acid sequence was changed from T to P. Thus, *Bch05G003700* was most likely the causal gene controlling green flesh. To further reveal the association between the SNP of *Bch05G003700* and green flesh, we cloned *Bch05G003700* of 45 chieh-qua germplasms from different genetic backgrounds, including commercial hybrids and inbred lines. As a result, homozygous C had white flesh, while homozygous A had green flesh.

Previous studies have revealed candidate genes for several important traits in wax gourd and chieh-qua, including fruit shape (Cheng et al., 2021), fruit peel color (Ma et al., 2021), fruit cuticular wax (Yan et al., 2022), seed shape (Luo et al., 2022), seed size (Yang et al., 2023), and gyneocy (Wang et al., 2023), and molecular markers have been developed for marker-assisted breeding (Huang et al., 2022). Interestingly, the locus controlling the peel color was also located on chromosome 5, and the causal gene *BhAPRR2* (*Bch05G003950*) is closely related to *CqLOL1* (*Bch05G003700*) with a physical distance of 1.11 Mb, which indicates that the two genes have a certain degree of linkage. Particularly, the chieh-qua fruits with green flesh always have pale green peel—for example, FJ3211 has green flesh and light green peel, and the linkage between green flesh and light green peel was observed in the F_2_ population. In this study, a dCAPS marker based on a point mutation in *CqLOL1* was developed to test the concordance of genotypes and phenotypes among 45 extreme materials, the result of which shows a complete correlation. The InDel marker Fc_13, close to *CqLOL1*, also showed an accuracy level of 100%. Thus, the two markers are useful for breeding the green flesh varieties of chieh-qua in the future.

On the other hand, the regulation of chlorophyll, including chlorophyll biosynthesis and degradation, is complex—for example, several genes involved in chlorophyll biosynthesis in rice, including *OsCHLH*, *OsCHLD*, *OsCHLI*, *OsCHLG*, *OsDVR*, *OsPOR*, and *OsCAO*, have been identified, and any mutations in these genes result in chlorophyll deficiency (Chen et al., 2018; Jung et al., 2003). Several *YGL* genes participate in chloroplast development, and these mutations lead to yellow green leaf in rice (Wu et al., 2007; Zhu et al., 2016). The key regulatory factors SGRs, PPH, and PAO that accelerate chlorophyll degradation have been identified in various species. In addition, several TFs, including NAC (Tak et al., 2018; Zou et al., 2023), HZP (Wu et al., 2024), TCP (Zheng et al., 2022), and MADS-box (Zhu et al., 2023) transcription factors, modulated SGRs, and *PPH* and *PAO* were regulated by MYB transcription factors (Wu et al., 2020). LSD1 is involved in programmed cell death, and it regulates photosynthesis-related genes, including PHANGs (photosynthesis-associated nuclear genes) and PHAPGs (photosynthesis-associated plastid genes), through its interaction with GOLDEN2-LIKE (GLK) transcription factors, thereby influencing the development and photosynthesis of chloroplasts (Lv et al., 2019; Li et al., 2022); in *Capsicum chinense*, the *CcLOL1* controls the immature fruit color, possibly by regulating chlorophyll synthesis genes such as POR, CAO, glutamyl-tRNA reductase (*HEMA1*), and the chlorophyll degradation gene pheophorbide oxygenase (Borovsky et al., 2019). The above-mentioned genes may be downstream genes of CqLOL1, and further efforts are needed to demonstrate it.

In conclusion, genetic analysis revealed that green flesh was a quantitative trait closely related to a normal distribution, and the *Cqgf* locus was first fine-mapped to a 329.70-kb region. By combining expression and sequence variation analysis, *CqLOL1* (*Bch05G003700*), which encodes the zinc-finger transcription factor LOL1, was proposed as the strongest candidate gene. Our findings not only provide a basis for marker-assisted selection but also help in understanding the molecular mechanism of green flesh formation.

## Data Availability

The data presented in the study are deposited in the Genome Sequence Archive in National Genomics Data Center, accession number CNP0005362.
