# Supplementary figures and images for: A point mutation in the zinc-finger transcription factor CqLOL1 controls the green flesh color in chieh-qua (Benincasa hispida Cogn. var. Chieh-qua How)
Source: Front Plant Sci. 2024 Oct 21;15:1388115. doi: 10.3389/fpls.2024.1388115 (PMC11532076; doi:10.3389/fpls.2024.1388115)

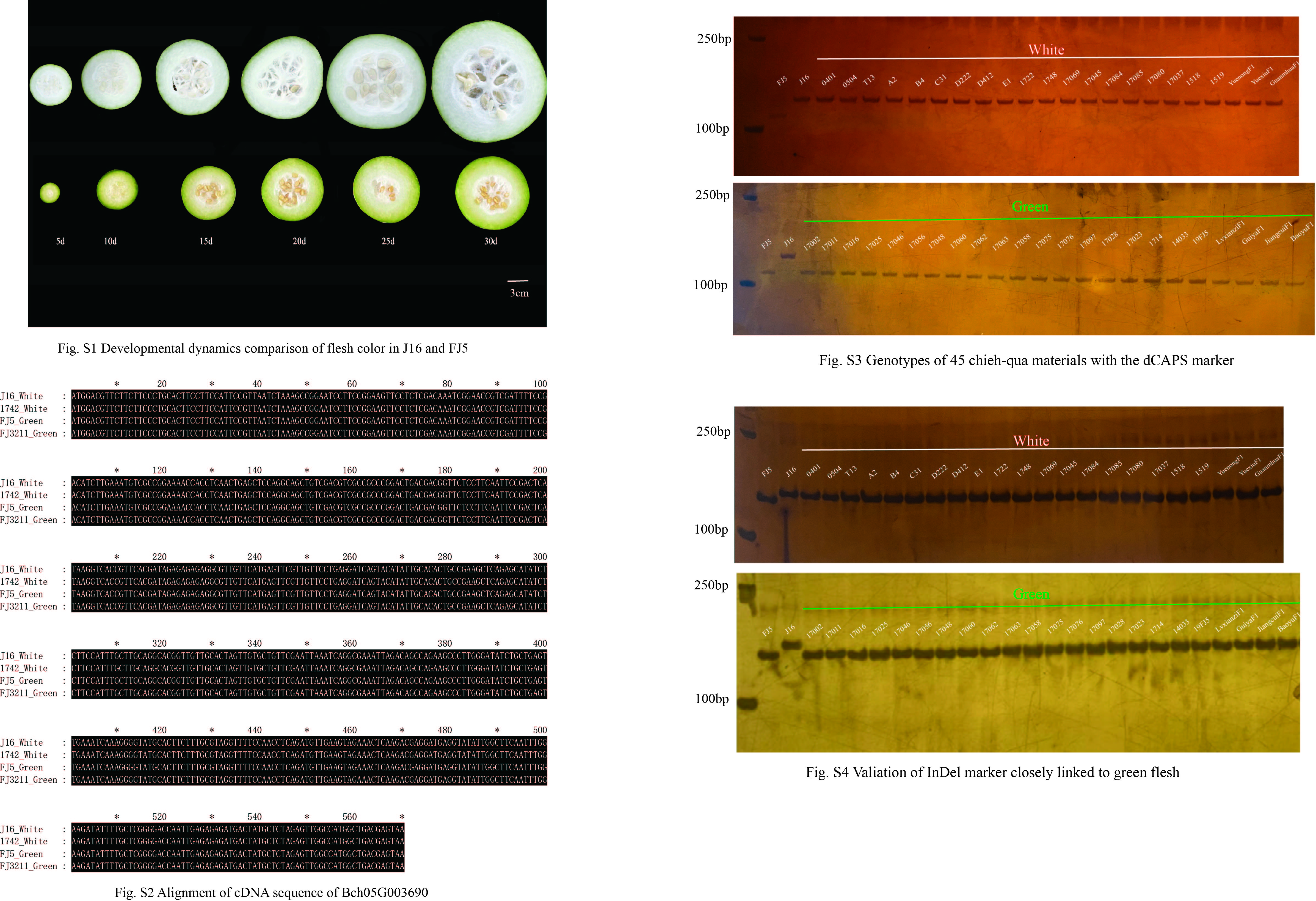

Supplement: Supplementary file 1 [file Image1.jpeg]
